# Current Study of the Mechanism of Action of the Potential Anti-Epileptic Agent Q808

**DOI:** 10.3390/molecules22071134

**Published:** 2017-07-07

**Authors:** Xiang Li, Hong-Jian Zhang, Qing Wang, Dian-Wen Zhang, Di Wu, Wei Li, Zhe-Shan Quan

**Affiliations:** 1 Key Laboratory for Molecular Enzymology and Engineering of Ministry of Education, College of Life Sciences, Jilin University, No. 2699 Qianjin Street, Changchun 130012, China; xiangl14@mails.jlu.edu.cn; 2 Key Laboratory of Natural Resources and Functional Molecules of the Changbai Mountain, Affiliated Ministry of Education, College of Pharmacy, Yanbian University, Yanji 133002, China; zhjzhishixiang@163.com (H.-J.Z.); zsquan@ybu.edu.cn (Z.-S.Q.); 3 Academy of Chinese Medical Sciences of Jilin Province, Changchun 130012, China; wangqingjy@163.com (Q.W.); zhangdianwenyao@163.com (D.-W.Z.); lizhaowu219@163.com (D.W.)

**Keywords:** Q808, anti-epileptic agent, neurotransmitters, mechanism, GABA

## Abstract

Our previous study showed that the anticonvulsant Q808 might be effective against seizures induced by maximal electroshock, pentylenetetrazole (PTZ), isoniazid (ISO), thiosemicarbazide (THIO), and 3-mercaptopropionic acid (3-MP). In the present study, we explored the possible mechanism of action of Q808. Results obtained with high-performance liquid chromatography (HPLC) suggest that Q808 may affect neurotransmitter content in the brain, by specifically increasing GABA content in the rat hippocampus at doses of 40 mg/kg and 80 mg/kg, and by reducing the content of glutamate and glutamine in the rat thalamus at a dose of 80 mg/kg. Intriguingly, there were no changes in the neurotransmitter content in the cortex in response to Q808. In vitro brain slice electrophysiological studies showed that 10^−5^ M Q808 enhanced the frequency of spontaneous inhibitory postsynaptic currents (sIPSCs) in corn cells of the CA1 area of the hippocampus, and had no effect on the amplitude of sIPSCs, the frequency and amplitude of spontaneous excitatory postsynaptic currents (sEPSCs), or γ-aminobutyric acid (GABA) receptor-mediated currents in primary cultured hippocampal neurons. These findings suggest that the antiepileptic activity of Q808 may be due to its ability to increase the amount of GABA between synapses, without affecting the function of GABA receptors.

## 1. Introduction

Epilepsy is a common neurological disorder that affects more than 60 million individuals worldwide annually [1]. Although epileptic seizures have been investigated for many years, the mechanisms underlying epileptogenesis are still unclear. Given this lack of knowledge, few treatment options are available to patients with epilepsy [2], and around one-third of patients are not satisfied with current pharmacotherapies [3]. Furthermore, the mechanisms of action of current anti-epilepsy drugs are diverse. For example, bumetanide displays anti-epileptic activity by acting on the GABA equilibrium potential of neurons or abnormal mature neurons, shifting the balance from depolarization to hyperpolarization, whereas cannabidiol exerts its antiepileptiform effects on both 4-aminopyridine-and Mg^2+^-free-induced epileptiform local field potentials [4,5,6,7,8]. Furthermore, ganaxolone plays a role on GABA_A_ receptors [9], whereas huperzine A is a potent acetylcholine esterase (AChE) inhibitor that increases GABAergic inhibition of abnormal CNS activity via pre-synaptic mechanisms [10].

**Figure 1:**
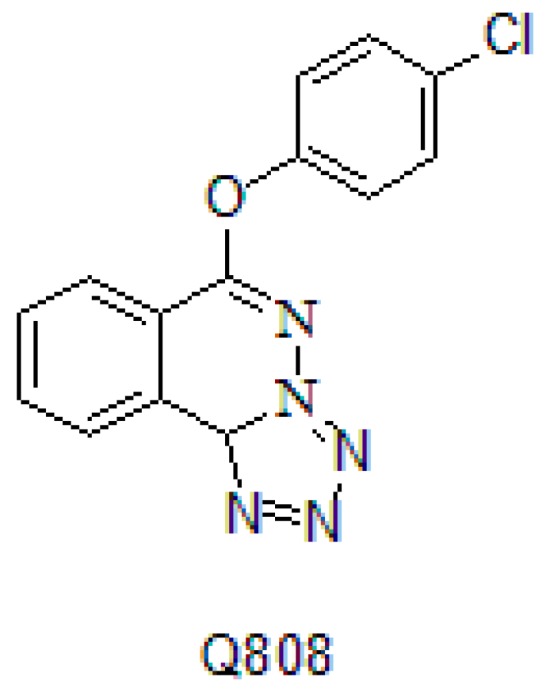
The chemical structure of Q808.

In our previous study, Q808 (6-(4-chlorophenoxy)-tetrazolo[5,1-*a*]phthalazine) exhibited potent anti-epileptic activity in a maximal electroshock mouse seizure model, with an ED_50_ of 6.8 mg/kg and a TD_50_ of 456.4 mg/kg. The chemical structure of Q808 is shown in Figure 1. Mice treated with Q808 were also resistant to seizures induced by PTZ, ISO, THIO, and 3-MP, with ED_50_ values of 22.8, 9.5, 2.2, and 1.5 mg/kg, respectively [11].

Different compounds may induce seizures via different mechanisms. It is thought that PTZ and ISO inhibit GABA neurotransmission [12,13]. However, THIO and 3-MP inhibit glutamate decarboxylase (GAD) [14]. In this study, we explored the possible mechanisms of action of the antiepileptic activity mediated by Q808.

## 2. Results

### 2.1. The Effect of Q808 on Neurotransmitters in the Brain

**Figure 2:**
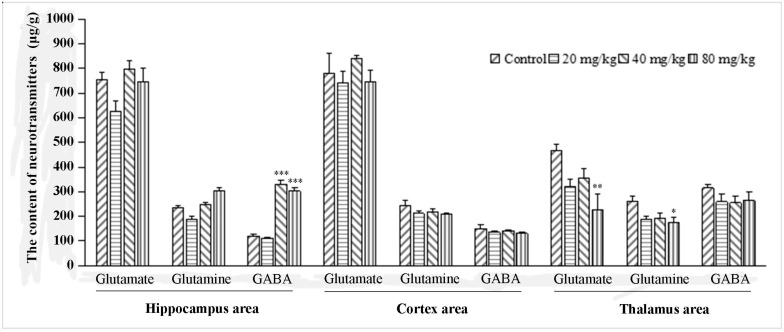
The effect of Q808 on neurotransmitters in the brain. * *p* < 0.05, ** *p* < 0.01, and *** *p* < 0.001 as compared to control.

Neurotransmitter measurements after Q808 administration are shown in Figure 2. In the hippocampal region, Q808 had no effect on the content of glutamate and glutamine. The content of GABA, however, was significantly increased by 40 mg/kg and 80 mg/kg doses of Q808. The largest amount of GABA was measured at 330.7 μg/g after a dose of 40 mg/kg Q808. Compared with the control group, there were no changes in the content of neurotransmitters in the cortical region. In the thalamic region, however, Q808 reduced neurotransmitter levels, as compared to control animals. More specifically, there was a significant difference in glutamate between the control group (465.0 μg/g) and rats that received 80 mg/kg Q808 (224.9 μg/g; *p* < 0.01), as well as a significant difference in glutamine between the control group (314.8 μg/g) and rats that received 80 mg/kg Q808 (174.8 μg/g; *p* < 0.05).

### 2.2. The Effect of Q808 on Electrophysiology in a Seizure Model

#### 2.2.1. The Influence of Q808 on Bicuculline-Induced Epileptiform Bursts

As shown in Table 1, there was a clear change in the frequency and amplitude of bicuculline-induced epileptiform bursts. The frequency was reduced from 1.424 Hz to 0.008 Hz when hippocampal slices were treated with 10^−4^ M Phenytoin. The rate of decrement was 0.987, which differed significantly from the control group (*p* < 0.001). Different doses of Q808 also reduced the frequency of epileptiform bursts in neurons. There was a dose-dependent effect on epileptiform bursts. Q808 yielded its greatest effect at a concentration of 10^−5^ mM, with a rate of decrement of 0.829 (*p* < 0.01). Intriguingly, although treatment with Q808 effectively reduced the frequency and amplitude, it was not as effective as phenytoin at inhibiting neuronal action potentials.

#### 2.2.2. The Influence of Q808 on High-K^+^-Induced Epileptiform Activity

The frequency of action potentials after treatment of CA1 pyramidal neurons with different concentrations of Q808 (10^−6^ M, 10^−5^ M and 10^−4^ M) is shown in Table 2. The frequency of action potentials was reduced from 0.579 Hz to 0.05 Hz after treatment with 10^−4^ M phenytoin (*p* < 0.01). The three concentrations of Q808 also reduced the frequency of neuronal action potentials, although treatment with 10^−6^ M Q808 did not yield a significant effect. Intriguingly, the efficiency of 10^−5^ M Q808 was similar to that of 10^−4^ M phenytoin. Neither phenytoin nor Q808 had an effect on the amplitude of neuronal action potentials.

#### 2.2.3. The Influence of Q808 on Epileptiform Activity Induced by Mg^2+^ Depletion

Application of Q808 also affected low-Mg^2+^-induced epileptiform activity. As shown in Table 3, the frequency of nerve impulses was reduced to 0.6703 Hz and 0.576 Hz when treated with Q808 at concentrations of 10^−5^ M and 10^−4^ M, respectively. The efficiency of Q808 increased as the concentration increased. The rates of decrement were 0.513 ± 0.122 (10^−6^ M, *n* = 8, *p* < 0.05), 0.659 ± 0.106 (10^−5^ M, *n* = 8, *p* < 0.05), and 0.802 ± 0.112 (10^−4^ M, *n* = 9, *p* < 0.01). There was no difference in amplitude between pre-and post-treatment in the experimental groups.

#### 2.2.4. The Influence of Q808 on sEPSCs and sIPSCs in the CA1 Region

**Figure 3:**
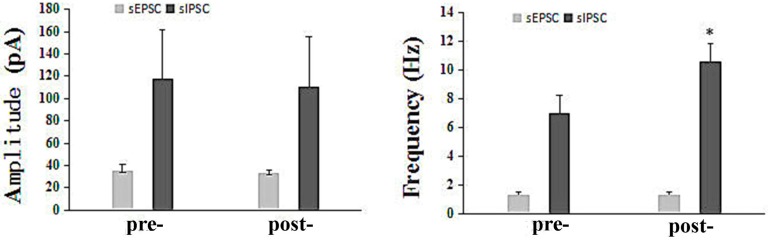
The effect of Q808 on sEPSC s and sIPSCs. * *p* *<* 0.05 as compared to pre-.

The effect of 10^−5^ M Q808 on sEPSCs and sIPSCs in the CA1 region is presented in Figure 3. The sEPSCs and sIPSCs were measured in six and nine pyramidal neurons in rat hippocampal slices, respectively. The mean amplitude of the sEPSCs was reduced from 35.05 pA to 33.31 pA, and the frequency was reduced from 1.359 Hz to 1.288 Hz after treatment with Q808. Similarly, the mean amplitude of the sIPSCs, was reduced from 117.9 ± 44.24 pA to 110.4 ± 45.34 pA after treatment with Q808. Neither of these differences, however, was significant. On the contrary, there was a significant increase in the average frequency upon treatment with Q808 (7.036 Hz to 10.64 Hz; *p* < 0.05).

### 2.3. The effect of Q808 on GABA Receptor-Mediated Current Characteristics

To investigate whether Q808 acts on GABA receptors, neuronal GABA receptor currents (*n* = 12) were measured and are shown in Table 4. Neurons in the experimental group were treated with a solution containing 50 μM GABA and 10 μM Q808, and neurons in the control group were treated with the same solution without Q808. The mean amplitude was 219.5 pA, which was slightly lower than that of the control group. There were no noticeable changes in receptor current amplitudes and decay times when compared to the control group.

## 3. Discussion

Previous studies have shown that some neurotransmitters, including glutamate, glutamine, and GABA, play an important role in epileptic seizures [15,16,17,18], and that many anti-epileptic drugs mediate their effects through their receptors. Thus, to determine the mechanism of action of Q808, it was worthwhile to measure changes in neurotransmitters. As our results indicated, Q808 significantly altered neurotransmitter levels in the hippocampus, as well as in the thalamus, but not in the cortex. This suggests that the anti-epileptic effect of Q808 can vary and is specific to certain brain regions. Hence, we hypothesized that its underlying mechanism was related to the GABAergic system in certain brain regions, including, most significantly, the hippocampus.

Bicuculline can induce epileptiform bursts by acting as a GABA_A_ receptor antagonist to block inhibitory synaptic transmission, which results in pyramidal cell depression [19,20]. We recorded changes in neuronal action potentials in hippocampal slices, and our results indicated that Q808 prevented epileptic seizure activity caused by inhibition of GABA_A_ receptors. This provided further evidence that its mechanism of action is associated with the GABAergic system. The efficacy of Q808 was not, however, dose-dependent. Based on the above reasons, we concluded that the GABAergic system might be one of several potential targets for Q808. It is likely that its mechanism is complex and involves other pathways in addition to the GABAergic system.

In order to investigate the possible mechanism of Q808, other electrophysiological experiments were performed. In an epileptiform model in which seizures were induced by high-K^+^ [21,22], a high concentration of exogenous K^+^ ions disturbs the balance of K^+^ inside and outside the cell membrane, which closes outwardly rectifying potassium ion channels, leading to depolarization and enhanced excitability. In this experiment, Q808 significantly reduced the frequency of neuronal action potentials, which suggests that it may be increasing the concentration of K^+^ ions or inactivating K^+^ channels. Furthermore, we suspect that Q808 may affect K^+^ channels; therefore, future studies should examine the influence of Q808 on both K^+^ and Na^+^ channels. In another epileptiform model in which seizures are induced by Mg^2+^ depletion, glutamate *N*-Methyl-*d*-aspartate (NMDA) receptors are no longer blocked by Mg^2+^, which results in the opening of their channels and enhanced excitatory synaptic transmission. Q808 was able to effectively suppress the frequency of pyramidal cell action potentials, and its efficiency was enhanced as its concentration was increased. Moreover, its effect was greater than that of the drug phenytoin. These results indicate that Q808 can intervene in epileptiform seizures caused by excessive opening of NMDA receptors, and that it likely has an inhibitory effect on these receptors.

Considering the complexity of its mechanism, we also investigated the effect of Q808 on GABA receptors and inhibitory synaptic transmission. These findings make important contributions toward the elucidation of the possible mechanisms of Q808. sEPSCs are spontaneous excitatory synaptic transmissions that are mediated by glutamate receptors [23,24], while sIPSCs are spontaneous inhibitions of synaptic transmission that are mediated by GABA receptors [25]. Our observations showed that Q808 altered the frequency of sIPSCs, without affecting the amplitude of sIPSCs or the frequency and amplitude of sEPSCs. We concluded that Q808 displayed anti-epileptic activity by increasing the frequency of GABA released at the synapse. When we measured GABA-mediated neuronal currents, we found no significant effect of treatment with Q808. This indicates that Q808 does not influence the function of GABA-related receptors.

## 4. Materials and Methods

### 4.1. Animals and Drugs

Sprague–Dawley rats (weight 280–340 g, half male and half female) were purchased from Zhejiang academy of medical sciences, China. Grade II, Certificate No. SCXK-2008-0033. They were maintained in individual cages with a 12-h light/dark cycle with free access to water and food. Q808 was prepared in our laboratory. Drugs and reagents were purchased from Sigma-Aldrich Chemical Company (St. Louis, MS, USA). All animal use protocols were approved by the local Institutional Animal Care and Use Committee.

### 4.2. The Maximal Electroshock Mouse Model of Epilepsy

Seizures were elicited in mice using a 60 Hz alternating current of 50 mA. The current was applied via corneal electrodes for 0.2 s. Tonic hind leg extension was regarded as a successful model of epilepsy. Protection against the spread of MES-induced seizures was defined as the abolition of tonic hind leg extension.

### 4.3. Measurement of Neurotransmitter Content of the Brain

Twenty normal male rats were randomly segregated into four equal groups. Solid dispersion particles containing Q808 were suspended in 0.5% CMC-Na (0.5 mL/100 g). The appropriate dose of the suspension liquid (20 mg/kg, 40 mg/kg, 80 mg/kg) was administered to rats via oral gavage. At 2 h after treatment with the suspension liquid, male rats were euthanized and decapitated. The cortex, thalamus, and hippocampus were subsequently dissected out on an ice-cold stainless steel plate. The brain tissue was homogenized in 0.1 M perchloric acid (10 μL/mg) and centrifuged at 15,000 rpm for 30 min at 4 °C. The supernatant was then collected and centrifuged at 15,000 rpm for 15 min and then re-collected and filtered with a 0.22 μm polyvinylidene difluoride membrane.

The contents of the cortex, thalamus, and hippocampus were separated using HPLC and measured with an electrochemical detector [26]. Derivating agent was prepared as a solution of 27 mg o-phthalaldehyde (OPA) dissolved in 1 ml methanol that was added to 10 mg thiofluor and 9 mL 100 M sodium tetraborate. The analytes were separated on a 3 μm, 3 × 50 mm Capcell Pak MGC18 column from Shiseido (Tokyo, Japan). The mobile phase was divided into two-components: component A (288 mL acetonitrile, 512 mL methanol, and 1200 mL 0.1 M Na_2_HPO_4_ aqueous solution) and component B (36 mL acetonitrile, 64 mL methanol, and 1900 mL 0.1 M Na_2_HPO_4_ aqueous solution). A gradient elution profile was used as follows: 0.00 min, isocratic 100% B; 10.00 min, isocratic 90% B; 20.00 min, isocratic 40% B; 30.00 min, isocratic 0% B; 35.00 min, isocratic 0% B; 36.00 min, isocratic 0% B; 41.00 min, isocratic 100% B. The flow rate was set to 0.7 mL/min. The temperature of the column was maintained at 38 °C. The data were acquired and analyzed using CoulArray software (ESA, Chelmsford, MA, USA).

### 4.4. Electrophysiological Model

Rat hippocampal slices were prepared as previously reported [27,28,29,30,31]. All efforts were made to minimize the number of animals used and their suffering. Male rats were anesthetized with ether and decapitated. Whole brains were isolated and stored in ice-cold artificial cerebrospinal fluid (ACSF) aerated with 95% O_2_/5% CO_2_. A polyethylene cannula was inserted in the basilar artery to restore brain perfusion with a solution composed of 120 mM NaCl, 25 mM NaHCO_3_, 3.3 mM KCl, 1.2 mM NaH_2_PO_4_, 10 mM glucose, 1.2 mM MgSO_4_, and 1.8 mM CaCl_2_. The rat hippocampi were sliced on a vibratome (LEICA VT1000 S, LEICA, Wetzlar, Germany) at 34 °C. Micropipettes were filled with ACSF and placed in the CA1 pyramidal cell layers. Action potentials of the CA1 pyramidal neuron were recorded using whole-cell patch clamp techniques (HEKA EPC10, HEKA Instruments, New York, NY, USA). Bicuculline ACSF was prepared by adding 50 μM bicuculline (Sigma, Shanghai, China) to ACSF. High-K^+^ ACSF consisted of 125 mM NaCl, 26 mM NaHCO_3_, 8 mM KCl, 1.2 mM CaCl_2_, 1.0 mM MgCl_2_, 1.25 mM NaH_2_PO_4_, and 10 mM glucose. Mg^2+^-free ACSF was prepared by omitting MgCl_2_ from the ACSF solution. Slices were incubated for 60 min prior to any experimental procedures. The sEPSCs and sIPSCs were measured as previously described [23,24]. Spontaneous postsynaptic currents (sEPSCs) in CA1 pyramidal cells were measured with whole-cell patch clamp techniques (HEKA EPC10, HEKA Instruments, New York, NY, USA).

### 4.5. Evaluation of the Effect of Q808 on GABA Receptor-Mediated Current Characteristics

Neuronal cultures were prepared from the hippocampi of Sprague–Dawley rats and were conducted as described by Huettner & Baughmann [32]. Donor mother rats were anesthetized with ether and rat embryos were dissected out and rapidly decapitated. The brains were removed, followed by dissection of the hippocampi. Neurons were then cultured on slides. To measure the current characteristics of the neurons, slides were placed in extracellular fluid at 25 °C, and whole cell membrane currents were measured with a two-electrode voltage-clamp. GABA receptor-mediated currents were measured after the addition of 50 μM exogenous GABA with an external spraying pipe. Each stimulus was repeated at 1-min intervals until two stable current signals were recorded, following by the addition of a solution consisting of 50 μM GABA and 10 μM Q808.

### 4.6. Statistical Analyses

Statistical analysis was carried out using SPSS 16.0 for Windows. Data are expressed as mean ± SEM. A one-way ANOVA and LSD test were used to compare the content of neurotransmitters in each area of the brain. A paired-samples *t*-test was used to compare measurements obtained before and after drug delivery, and an independent-samples t-test was used to compare GABA receptor-mediated current characteristics.

## 5. Conclusions

To summarize, the present results indicate that the underlying mechanism of Q808 is associated with the GABAergic system in certain brain regions. Q808 may enhance the frequency of sIPSCs and increase the content of GABA in the hippocampus without affecting the function of GABA-related receptors. Moreover, Q808 may have a direct effect on NMDA receptors.

## Figures and Tables

**Table 1:** The effect of Q808 on bicuculline-induced epileptiform burst.

Groups		Control	Phenytoin	Q808		
Concentration		- (*n* = 5) ^a^	10^−4^ M (*n* = 6)	10^−6^ M (*n* = 8)	10^−5^ M (*n* = 10)	10^−4^ M (*n* = 9)
Frequency (Hz)	pre- ^b^	4.247 ± 1.063	1.424 ± 0.539	1.646 ± 0.243	3.400 ± 0.875	2.169 ± 0.754
	post- ^b^	4.194 ± 1.437	0.008 ± 0.008 *	0.385 ± 0.116 **	0.337 ± 0.148 **	0.947 ± 0.291 *
Amplitude (mV)	pre- ^b^	27.09 ± 0.833	25.614 ^c^	34.070 ± 5.288	34.310 ± 3.937	44.140 ± 5.232
	post- ^b^	29.85 ± 4.161	28.514 ^c^	33.980 ± 5.431	22.590 ± 7.328	33.000 ± 5.169
Frequency rate of decrement		0.088 ± 0.089	0.987 ± 0.013 ^###^	0.696 ± 0.111 ^###^	0.829 ± 0.0729 ^##^	0.590 ± 0.071 ^###^

* *p* *<* 0.05, **^, ##^ *p* *<* 0.01, and ^###^ *p* *<* 0.001 as compared to control; ^a^ The number of observed neuron, - : not treatment; ^b^ pre-: pre-treatment; post-: post-treatment; ^c^ only one neuron could be observed the changes of action potential per- and post-treatment with phenytoin.

**Table 2:** The effect of Q808 on high-K^+^-induced epileptiform activity.

Groups		Control	Phenytoin	Q808		
Concentration		- (*n* = 5)	10^−4^ M (*n* = 7)	10^−6^ M (*n* = 9)	10^−5^ M (*n* = 9)	10^−4^ M (*n* = 10)
Frequency (Hz)	pre-	2.663 ± 0.943	0.579 ± 0.155	0.821 ± 0.190	1.462 ± 0.377	0.794 ± 0.169
	post-	1.800 ± 0.416	0.05 ± 0.024 **	0.336 ± 0.148 **	0.139 ± 0.031 **	0.270 ± 0.110
Amplitude (mV)	pre-	34.84 ± 1.290	57.60 ± 11.260	55.370 ± 6.842	58.980 ± 6.259	51.060 ± 6.335
	post-	34.61 ± 1.741	67.400 ± 18.00	53.46 ± 5.747	52.14 ± 6.728	49.71 ± 11.39
Frequency rate of decrement		0.183 ± 0.127	0.921 ± 0.030	0.665 ± 0.096	0.817 ± 0.078	0.283 ± 0.345

** *p* *<* 0.01 as compared to control; - : not treatment.

**Table 3:** The effect of Q808 on low-Mg^2+^-induced epileptiform activity.

Groups		Control	Phenytoin	Q808		
Concentration		- (*n* = 6)	10^−4^ M (*n* = 9)	10^−6^ M (*n* = 8)	10^−5^ M (*n* = 8)	10^−4^ M (*n* = 9)
Frequency (Hz)	pre-	1.583 ± 0.391	1.003 ± 0.229	1.233 ± 0.409	1.726 ± 0.398	1.810 ± 0.465
	post-	1.527 ± 0.519	0.372 ± 0.241 **	0.441 ± 0.180	0.6703 ± 0.257 *	0.576 ± 0.241 *
Amplitude (mV)	pre-	35.34 ± 0.960	37.850 ± 0.167	43.530 ± 5.220	37.730 ± 3.874	34.110 ± 3.490
	post-	40.53 ± 0.060	32.810 ± 1.510	34.380 ± 9.550	35.750 ± 2.062	31.750 ± 3.360
Frequency rate of decrement		0.060 ± 0.145	0.183 ± 0.127	0.513 ± 0.122 ^#^	0.659 ± 0.106 ^#^	0.802 ± 0.112 ^##^

*^,#^ *p* *<* 0.05 and **^,##^ *p* *<* 0.01 as compared to control; - : not treatment.

**Table 4:** The effect of Q808 on GABA receptor-mediated current characteristics (*n* = 12).

Groups	Control	Q808
Amplitude (pA)	227.3 ± 62.00	219.5 ± 52.55
The time to half-decay (↑ ^a^, ms)	661.0 ± 47.91	575.4 ± 38.86
The time to half-decay (↓ ^b^, ms)	11220 ± 2767	10360 ± 1882

^a^ Increasing current; ^b^ Dropping current.

## References

[1] Strine T.W., Kobau R., Chapman D.P., Thurman D.J., Price P., Balluz L.S. (2005). Psychological distress, comorbidities, and health behaviors among U.S. adults with seizures: Results from the 2002 National Health Interview Survey. Epilepsia.

[2] Ionov I.D. (2009). Self-reinforcing loop mechanism in epilepsy. Med. Hypotheses.

[3] Löscher W. (2002). Current status and future directions in the pharmacotherapy of epilepsy. Trends Pharmacol. Sci..

[4] Dzhala V.I., Talos D.M., Sdrulla D.A., Brumback A.C., Mathews G.C., Benke T.A., Delpire E., Jensen F.E., Staley K.J. (2005). NKCC1 transporter facilitates seizures in the developing brain. Nat. Med..

[5] Rheims S., Minlebaev M., Ivanov A., Represa A., Khazipov R., Holmes G.L., Ben-Ari Y., Zilberter Y. (2008). Excitatory GABA in rodent developing neocortex in vitro. J. Neurophysiol..

[6] Ben-Ari Y., Khalilov I., Kahle K.T., Cherubini E. (2012). The GABA excitatory/inhibitory shift in brain maturation and neurological disorders. Neuroscientist.

[7] Löscher W., Puskarjov M., Kaila K. (2013). Cation-chloride cotransporters NKCC1 and KCC2 as potential targets for novel antiepileptic and antiepileptogenic treatments. Neuropharmacology.

[8] Jones N.A., Hill A.J., Smith I., Bevan S.A., Williams C.M., Whalley B.J., Stephens G.J. (2010). Cannabidiol displays antiepileptiform and antiseizure properties in vitro and in vivo. J. Pharmacol. Exp. Ther..

[9] Carter R.B., Wood P.L., Wieland S., Hawkinson J.E., Belelli D., Lambert J.J., White H.S., Wolf H.H., Mirsadeghi S., Tahir S.H. (1997). Characterization of the anticonvulsant properties of ganaxolone (CCD 1042; 3alpha-hydroxy-3beta-methyl-5alpha-pregnan-20-one), a selective, high-affinity, steroid modulator of the gamma-aminobutyric acid(A) receptor. J. Pharmacol. Exp. Ther..

[10] Tang X.C., Han Y.F. (2009). Pharmacological profile of huperzine A, a novel acetylcholinesterase inhibitor from Chinese herb. CNS Drug Rev..

[11] Sun X.Y., Wei C.X., Deng X.Q., Sun Z.G., Quan Z.S. (2010). Evaluation of the anticonvulsant activity of 6-(4-chlorophenyoxy)-tetrazolo[5,1-*a*]phthalazine in various experimental seizure models in mice. Pharmacol. Rep..

[12] Okada R., Negishi N., Nagaya H. (1989). The role of the nigrotegmental GABAergic pathway in the propagation of pentylenetetrazol-induced seizures. Brain Res..

[13] Olsen RW. (1981). GABA-benzodiazepine-barbiturate receptor interactions. J. Neurochem..

[14] Löscher W. (1979). 3-Mercaptopropionic acid: Convulsant properties, effects on enzymes of the gamma-aminobutyrate system in mouse brain and antagonism by certain anticonvulsant drugs, aminooxyacetic acid and gabaculine. Biochem. Pharmacol..

[15] Sun H.L., Zhu W., Zhang Y.R., Pan X.H., Zhang J.R., Chen X.M., Liu Y.X., Li S.C., Wang Q.Y., Deng D.P. (2017). Altered glutamate metabolism contributes to antiepileptogenic effects in the progression from focal seizure to generalized seizure by low-frequency stimulation in the ventral hippocampus. Epilepsy Behav..

[16] Kanamori K. (2015). Disinhibition reduces extracellular glutamine and elevates extracellular glutamate in rat hippocampus in vivo. Epilepsy Res..

[17] Van der Hel W.S., Hessel E.V., Bos I.W., Mulder S.D., Verlinde S.A., van Eijsden P., de Graan P.N. (2014). Persistent reduction of hippocampal glutamine synthetase expression after status epilepticus in immature rats. Eur. J. Neurosci..

[18] DiNuzzo M., Mangia S., Maraviglia B., Giove F. (2014). Physiological bases of the K^+^ and the glutamate/GABA hypotheses of epilepsy. Epilepsy Res..

[19] Galzigna L., Garbin L., Bianchi M., Marzotto A. (1978). Properties of two derivatives of gamma-aminobutyric acid (GABA) capable of abolishing Cardiazol^−^ and bicuculline-induced convulsions in the rat. Arch. Int. Pharmacodyn. Ther..

[20] Lu H.C., Chang W.J., Chang W.P., Shyu B.C. (2016). Direct-current Stimulation and Multi-electrode Array Recording of Seizure-like Activity in Mice Brain Slice Preparation. J. Vis. Exp..

[21] Yechikhov S., Shchipakina T., Savina T., Kalemenev S., Levin S., Godukhin O. (2002). The role of Ca^2+^/calmodulin-dependent protein kinase II in mechanisms underlying neuronal hyperexcitability induced by repeated, brief exposure to hypoxia or high K^+^ in rat hippocampal slices. Neurosci. Lett..

[22] Liu J.S., Li J.B., Gong X.W., Gong H.Q., Zhang P.M., Liang P.J., Lu Q.C. (2013). Spatiotemporal dynamics of high-K^+^-induced epileptiform discharges in hippocampal slice and the effects of valproate. Neurosci. Bull..

[23] Chavez-Noriega L.E., Stevens C.F. (1994). Increased transmitter release at excitatory synapses produced by direct activation of adenylate cyclase in rat hippocampal slices. J. Neurosci..

[24] Vignes M. (2001). Regulation of spontaneous inhibitory synaptic transmission by endogenous glutamate via non-NMDA receptors in cultured rat hippocampal neurons. Neuropharmacology.

[25] Tao W., Higgs M.H., Spain W.J., Ransom C.B. (2013). Postsynaptic GABAB receptors enhance extrasynaptic GABAA receptor function in dentate gyrus granule cells. J. Neurosci..

[26] Jin C.L., Yang L.X., Wu X.H., Li Q., Ding M.P., Fan Y.Y., Zhang W.P., Luo J.H., Chen Z. (2005). Effects of carnosine on amygdaloid-kindled seizures in Sprague-Dawley rats. Neuroscience.

[27] Duffy S., Nguyen P.V., Baker G.B. (2004). Phenylethylidenehydrazine, a novel GABA-transaminase inhibitor, reduces epileptiform activity in rat hippocampal slices. Neuroscience.

[28] Semyanov A., Godukhin O. (2001). Epileptiform activity and EPSP-spike potentiation induced in rat hippocampal CA1 slices by repeated high-K(+): Involvement of ionotropic glutamate receptors and Ca(2+)/calmodulin-dependent protein kinase II. Neuropharmacology.

[29] Bikson M., Id Bihi R., Vreugdenhil M., Kohling R., Fox J.E., Jefferys J.G. (2002). Quinine suppresses extracellular potassium transients and ictal epileptiform activity without decreasing neuronal excitability in vitro. Neuroscience.

[30] Jones R.S. (1989). Ictal epileptiform events induced by removal of extracellular magnesium in slices of entorhinal cortex are blocked by baclofen. Exp. Neurol..

[31] Sharopov S., Moser J., Chen R., Kolbaev S.N., Bernedo V.E., Werhahn K.J., Luhmann H.J., Kilb W. (2012). Dopaminergic modulation of low-Mg(2)(+)-induced epileptiform activity in the intact hippocampus of the newborn mouse in vitro. J. Neurosci. Res..

[32] Huettner J.E., Baughman R.W. (1986). Primary culture of identified neurons from the visual cortex of postnatal rats. J. Neurosci..

